# 

**Published:** 1906-05-12

**Authors:** 


CONTENTS.

**The Prolongation of Life** - - - - - - - - - - 97

**Annotations** - - - - - - - - - - - - 98

**Hospital Clinics—**

On the Causes and Treatment of Gangrene of the Leg - - 99

Stigmata of Degeneration and the Prognosis of Epilepsy - - 101

Discussion on Syphilis - - - - - - - - - 102

Gallant's Corsets in the Treatment of Enteroptosis - - - 103

**Progress in Medicine and Surgery—**

Diseases of the Blood - - - - - - - - 104

Diseases of Digestive Organs - - - - - - - - 104

**New Appliances and Things Medical** - - - - 106

**Medical Appointments and Vacancies** - - - - 106

**Hospital Administration—**

Motor Cars for Doctors - - - - - - - - - - 107

Current Hospital Topics - - - - - - - - - 109

South Staffordshire Hospital for Small-pox - - - - 110

Social and Poor Law Problems - - - - - - - - 112

The Bolingbroke Hospital - - - - - - - - - 112

Hospital Meetings - - - - - - - - - - - 113

**Notes and News** - - - - - - - - - - - 114

*NURSING SECTION.*

**Notes on News from the Nursing World—** The Princess Royal and the Children—Queen Victoria's Jubilee Institute—Death of an Army Matron—Progress of a great Trained Nurses' Institution—English Nurses and Prospects in Brussels—Another Case of Interference with a Matron—Dinner to an Australian Matron—The Work of the East End Mothers' Lying-in Home—Lady Londonderry on the Nurse and the Mother—Pauper Attendants at Midland Poor-law Infirmaries—Nursing under Christian Scientist Auspices—Dividing the Income of a Bequest—The Matronship of Brecon Infirmary—Disgraceful Assault on a Nurse at a London Infirmary—Invalid Cookery Examination at Wakefield—Short Items 85-88
**The Nursing Outlook** - - - - - - - - - 89
**Abdominal Surgery** - - - - - - - - - - 90
**The Nurses' Clinic** - - - - - - - - - - 91
**Incidents in a Nurse's Life** - - - - - - - - 92
**A Visit to the Midland and Birmingham Women's Hospital** - - - - - - - - 92
**Central Midwives Board** - - - - - - - - 93
**Rural Midwives' Association** - - - - - - - 94
**The Training and Registration of Asylum Nurses** 95
**Everybody's Opinion** - - - - - - - - - 95
**Appointments** - - - - - - - - - - - 96
**Death in Our Ranks** - - - - - - - - - 97
**Novelties for Nurses** - - - - - - - - - 97
**Notes and Queries** - - - - - - - - - - 98
**For Reading to the Sick** - - - - - - - - 98

